# Bacterial Communities of the Coronal Sulcus and Distal Urethra of Adolescent Males

**DOI:** 10.1371/journal.pone.0036298

**Published:** 2012-05-11

**Authors:** David E. Nelson, Qunfeng Dong, Barbara Van Der Pol, Evelyn Toh, Baochang Fan, Barry P. Katz, Deming Mi, Ruichen Rong, George M. Weinstock, Erica Sodergren, J. Dennis Fortenberry

**Affiliations:** 1 Department of Biology, Indiana University, Bloomington, Indiana, United States of America; 2 Department of Biological Sciences, Department of Computer Science & Engineering, University of North Texas, Denton, Texas, United States of America; 3 School of Public Health, Indiana University, Bloomington, Indiana, United States of America; 4 Department of Biostatistics, Indiana University School of Medicine, Indianapolis, Indiana, United States of America; 5 Department of Pediatrics, Indiana University School of Medicine, Indianapolis, Indiana, United States of America; 6 Department of Genetics, Washington University St. Louis School of Medicine, St. Louis, Missouri, United States of America; Institute for Genome Sciences, University of Maryland School of Medicine, United States of America

## Abstract

*Lactobacillus-*dominated vaginal microbiotas are associated with reproductive health and STI resistance in women, whereas altered microbiotas are associated with bacterial vaginosis (BV), STI risk and poor reproductive outcomes. Putative vaginal taxa have been observed in male first-catch urine, urethral swab and coronal sulcus (CS) specimens but the significance of these observations is unclear. We used 16 S rRNA sequencing to characterize the microbiota of the CS and urine collected from 18 adolescent men over three consecutive months. CS microbiotas of most participants were more stable than their urine microbiotas and the composition of CS microbiotas were strongly influenced by circumcision. BV-associated taxa, including *Atopobium*, *Megasphaera*, *Mobiluncus*, *Prevotella* and *Gemella*, were detected in CS specimens from sexually experienced and inexperienced participants. In contrast, urine primarily contained taxa that were not abundant in CS specimens. *Lactobacilllus* and *Streptococcus* were major urine taxa but their abundance was inversely correlated. In contrast, *Sneathia*, *Mycoplasma* and *Ureaplasma* were only found in urine from sexually active participants. Thus, the CS and urine support stable and distinct bacterial communities. Finally, our results suggest that the penis and the urethra can be colonized by a variety of BV-associated taxa and that some of these colonizations result from partnered sexual activity.

## Introduction

The bacterial communities (microbiotas) associated with different body surfaces can impact pathogen colonization resistance and autoimmune disease [1]. For example, dysbioses of the gastrointestinal tract microbiota can trigger overgrowth of pathogens [1] such as *C. difficile* [2], which are linked to chronic inflammatory conditions including Crohn’s disease and ulcerative colitis [3], [4], and can increase risk of colonization by enteropathogens including *Clostridia*, *Salmonella*, *Vibrio*, *Escherichia* and *Shigella* spp. [5], [6]. Probiotic activities of *Lactobacillus* spp. that colonize the vagina illustrate mechanisms by which the microbiota can influence susceptibility to infectious disease [7]. *Lactobacillus* spp. regulate the balance of pro-inflammatory cytokines in vaginal secretions [8], [9], [10], block colonization and invasion of some pathogens [11] and produce lactic acid, hydrogen peroxide [12] and bacteriocins [13] that inhibit other vaginal microorganisms. Reduction of vaginal *Lactobacillus* spp. is associated with the overgrowth of anaerobic bacteria that occurs in bacterial vaginosis (BV) [14], and increased susceptibility to bacterial and viral sexually transmitted infection (STI) [15], [16]. Thus there is strong evidence that the composition of the female reproductive tract microbiota is linked to reproductive health and resistance to STI in women.

In comparison, the microbiota of the male reproductive tract is poorly described. The penis itself provides distinct anatomical environments in the urethra and the coronal sulcus (CS). Both sites are exposed to similar foreign microbial communities during sexual activity. Some bacteria transferred during sexual activity (e.g., *Neisseria gonorrhoeae* and *Chlamydia trachomatis*) cause substantial world-wide morbidity [17]. In addition, the CS and distal urethras of healthy men at least episodically support bacterial communities [18], [19], [20], [21]. *Lactobacillus* spp. have been identified in urine and urethral swabs [19], and BV-associated taxa including *Prevotella*, *Gardnerella* and *Sneathia* are found in CS [20] and urethral specimens from adult men [18], [19]. Although the role of bacteria in the male urethra is unknown, the CS microbiota has been hypothesized to mediate effects of circumcision on risk of HIV and other STI [20].

A limitation in understanding the microbiota of the penis is the lack of data from healthy young men who have and or have not had partnered sexual experiences. These data would allow more thorough description of the microbiota of the urethra and CS, and could provide insight into changes associated with sexual exposures. To fill this gap, we collected urine and CS specimens from eighteen healthy 14–17 year old men with varied circumcision status and sexual histories. Sampling was repeated at monthly intervals to investigate stability of the microbiota over a three-month period. Bacteria were identified using multiple 16 S rRNA sequencing methods. Urine and corresponding CS specimens supported stable, but dissimilar microbiotas. Major urine taxa in most of the sexually experienced and inexperienced participants were members of the order *Lactobacillialles*. Finally, some bacteria were detected only in participants with histories of partnered sexual activity.

## Results and Discussion

### Compositions of CS and urine microbiotas are similarly measured by different 16 S rRNA sequencing approaches

There were no data concerning optimal methods for cultivation-independent characterization of CS or urine microbiotas; thus we analyzed total genomic DNA from 72 CS and urine specimens using four different 16 S rRNA sequencing approaches. Near full-length 16 S rRNA alleles were assembled from individual Sanger sequence reads [18], while the V1–V3, V3–V5 and V6–V9 sub-regions of 16 S rRNA alleles were analyzed by multiplex PCR and pyrosequencing using a protocol developed by human microbiome demonstration projects (http://hmpdacc.org) [22]. Only 8 urine and 3 CS specimens yielded poor quality or no PCR amplicons using the Sanger approach and these same specimens tended to yield poor pyrosequencing results. This indicated that some specimens either had low bacterial loads or contained inhibitors that blocked 16 S PCR amplification. Taxonomy was assigned using RDP classifier. In total, 56,406; 756,640; 759,914 and 1,212,918 sequences from full-length, V1–V3, V3–V5 and V6–V9 16 S rRNA regions were assigned to the genus level with 90% confidence, respectively (Tables S1, S2, S3, S4, S5).

**Figure 1:**
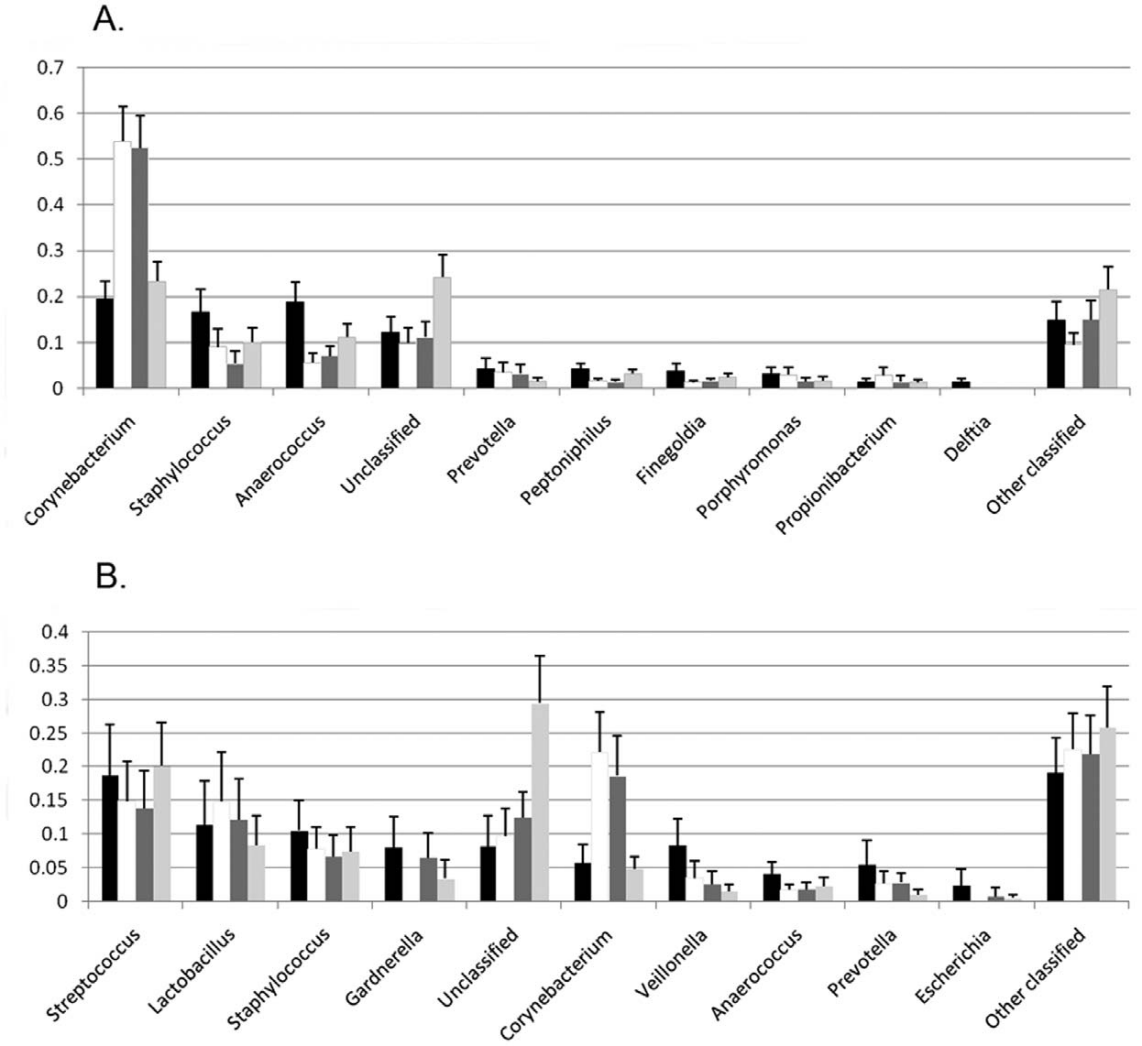
Comparison of CS and urine microbiotas measured by different 16 S rRNA sequencing methods. Distribution of RDP taxa (90% confidence) in enrollment (A) CS and (B) urine specimens. Proportions of normalized sequences are on the Y-axis. (black) Sanger, (white) V1–V3, (dark gray) V3–V5 and (light gray) V6–V9 sequence data sets.

PCR-based 16 S rRNA identification can introduce biases during priming, amplification, cloning and sequencing [23]. Types and proportions of taxa identified in urine and CS specimens by each sequencing approach were compared. Major taxa and their relative proportions were similar in all the datasets (Figures 1a and 1b; Tables S1, S2, S3, S4, S5) with the following exceptions: V6–V9 under-represented *Prevotella*, *Gardnerella* was not captured by the V1–V3 approach, and *Corynebacteria* were over-represented in both V1–V3 and V3–V5. These results indicated that sub-region sequencing provided reasonable coverage of both urethral and CS bacterial communities, with the caveat that validation against other methods is warranted to reveal taxa missed by any single method. Since a large number of near full-length Sanger 16 S sequences were obtained and pyrosequencing failed to reveal additional major taxa our subsequent analyses were based on the Sanger data and cross-checked against the sub-region data sets.

### The CS Supports a Complex and Stable Microbiota

**Figure 2:**
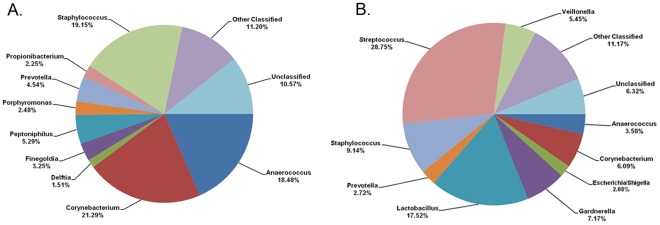
Distribution of major taxa in enrollment CS and urine specimens. Sanger data-set. (A) CS specimens. (B) urine specimens.

In the 17/18 enrollment specimens that yielded 16 S rRNA amplicons, 58 high confidence taxa were predicted from 9,070 16 S rRNA sequences (Figure 2A). Three genera were in most specimens; *Corynebacteria* (16/17), *Staphylococcus* (16/17) and *Anaerococcus* (15/17), and these accounted for more than 58.9% of the sequences. Other abundant genera, in order of relative abundance, included *Peptoniphilus* (13/17), *Prevotella* (4/17), *Finegoldia* (14/17), *Porphyromonas* (8/17), *Propionibacterium* (11/17) and *Delftia* (8/17). All of these genera, along with high quality sequences that could not be classified to the genus-level with 90% confidence (11.2%), accounted for an additional 30.5% of the sequences (Figure 2A and Table S1). *Pseudomonas* were notably less abundant (<0.01%) in CS specimens in this cohort than they were in a group of adult African men described in a previous study [20].

**Figure 3:**
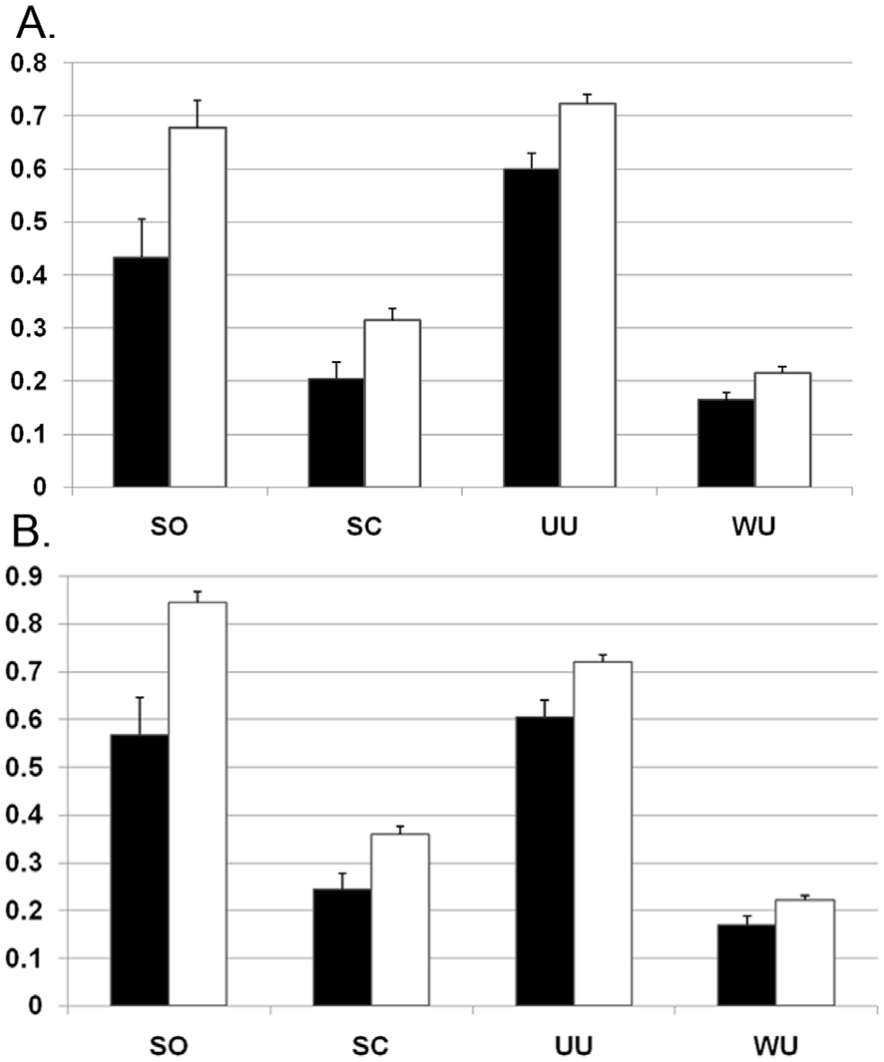
Comparison of intraperson and interperson similarity of CS and urine microbiotas. Black bars indicate comparison of specimens from the same participant (4 specimens) and white bars indicate comparison of each specimen to all specimens from different participants. A) CS specimens. B) Urine specimens. SO, Sørenson similarity index; SC, Spearman similarity coefficient; UU, unweighted Unifrac distance; WU, weighted Unifrac distance. Wilcoxon P-values for all comparisons (same versus different participants) were all <1 E 10^−6^. Bars indicate 95% confidence intervals.

**Figure 4:**
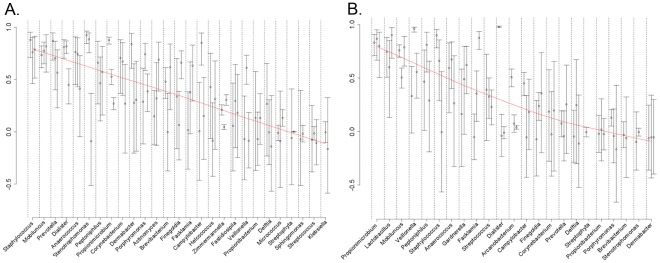
Temporal stability of CS and urine taxa . Lin’s concordance correlation coefficients (Y-axis) were calculated to assess agreement of abundance of taxa, (X-axis), in sequential (A) CS, or (B) urine specimens from the same participants (three intervals: months 0–1, 1–2, 2–3), the red trend line indicates mean of the three intervals. Bars indicate 95% confidence intervals.

Comparison of major taxa in enrollment and subsequent monthly CS specimens indicated no dramatic differences in composition during the study interval. To assess stability of the CS microbiota, weighted and unweighted Unifrac PCA [24], the Sørenson similarity index and Spearman correlation coefficient were used to compare longitudinal CS specimens. All of these measures confirmed that longitudinal CS specimens from the same participant were significantly more similar to one another than to CS specimens from other participants (Figure 3A). Lin’s concordance correlation coefficents were calculated to measure stability of individual taxa (Figure 4A). *Staphyloccoccus*, *Mobiluncus*, *Prevotella*, *Dialister* and *Anaerococcus* yielded mean values of >0.5, indicating that these taxa were stable members of CS communites. In contrast, major urine taxa including *Veillonella* and *Streptococcus* were detected in some CS specimens but yielded values between 0 and 0.25. This indicates that although some urine taxa periodically colonize or reside in the CS, they are not stable members of the CS microbiota.

### Circumcision alters the CS microbiota

**Figure 5:**
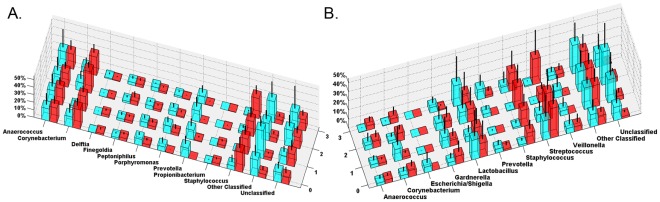
Impact of circumcision on the CS and urine microbiotas. Relative normalized abundance of major (A) CS and (B) urine taxa in circumcised (red) and uncircumcised (blue) participants at all four sampling points (Z-axis). Bars indicate 95% confidence intervals.

Price and colleagues reported that circumcision alters the adult CS microbiota and that anaerobic and putative vaginal taxa are most abundant prior to circumcision, whereas aerobes and skin taxa increase following circumcision [20]. Taxa in enrollment specimens from participants who had (12/17) and who had not (5/17) been circumcised were compared to test if similar relationships could be detected in young men (Figure 5A). Consistent with the Price study, *Corynebacteria* and *Anaerococcus* were dominant in both groups and were present in similar proportions. *Staphylococcus* was significantly enriched (26.6% vs. 5.5%) in enrollment specimens from circumcised participants (Wilcoxon signed-rank test, p-value = 0.0048) while *Porphyrmonas* was enriched (6.4% vs. 0.3%) in uncircumcised participants (Wilcoxon signed-rank test, p-value = 0.026). *Prevotella* was exclusively identified in uncircumcised participants (4/6) (Fisher’s exact test p-value = 0.006) and was abundant in this sub-group (12.9%).

**Figure 6:**
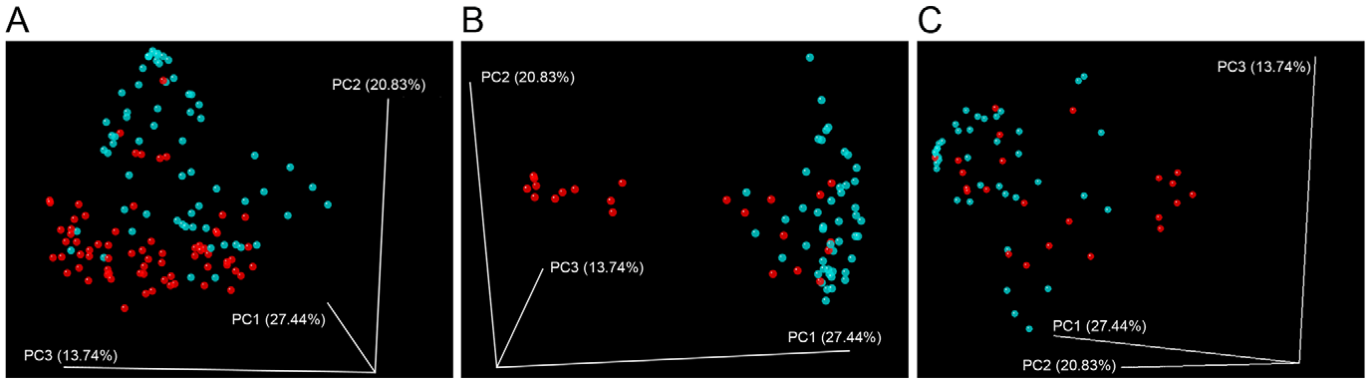
Similarity of CS and urine taxa . A-C) Weighted Unifrac comparison of the microbiotas in select groups of specimens (Sanger data-set). A) All CS (red) and all urine (blue) specimens. B) Circumcised (red) and uncircumcised (blue) CS specimens. C) Circumcised (red) and uncircumcised (blue) urine specimens.

Weighted Unifrac analysis robustly separated CS and urine specimens (Figure 6A). This analysis also clearly differentiated circumcised and uncircumcised CS specimens (Figure 6B). In contrast, groups of circumcised and uncircumcised urines were not as clearly differentiated (Figure 6C). Clustering of CS and urine CS specimens from each sampling point based upon Bray-Curtis and Sørenson’s similarity coefficients as a measure of distance also indicated that CS and urine specimens from the same subject rarely grouped together (Figure S1 and data not shown). Collectively, these results indicated that circumcision most strongly impacted the CS and that the microbiotas in corresponding CS and urine specimens were dissimilar.

Circumcised and uncircumcised CS contained taxa that have been associated with the superficial skin [25] and especially moist skin sites, such as the inguinal crease [26]. CS contained high proportions of *Corynebacteria* or *Staphyloccocus* and lower proportions of *Propionibacteria* and *Betaproteobacteria*; patterns associated with sebaceous and dry skin sites, respectively [26]. Some taxa including *Prevotella* and *Porphyromonas* were more abundant in uncircumcised CS, but were not predominant components of the CS microbiota. Interestingly, multiple taxa common in urine were rare or absent from CS specimens.

### Microbiotas of Urine and CS are Dissimilar

18/18 enrollment urines yielded specific PCR amplicons and 51 high confidence taxa were predicted from a total of 10,144 16 S rRNA sequences. Some abundant CS taxa were detected in urine but microbiotas of these specimen types were dissimilar (Figure 2). Urine contained high proportions of genera whose members are obligate and/or facultative anaerobes (Figure 2B). *Streptococcus* (11/18), *Lactobacillus* (4/18), *Gardnerella* (5/18) and *Veillonella* (4/18), accounted for 59.1% of enrollment urine sequences. Closest RDP matches of almost all of the enrollment *Lactobacillus and Gardnerella* sequences corresponded to *L. iners* and *G. vaginalis*, while more diverse species of *Streptococcus* (*agalactiae*, *mitis*, *anginosus* and uncultured taxa) and *Veillonella* (*dispar*, *atypica*, *montpellierensis*, *parvula* and uncultured taxa) were represented. In contrast, only 1.3% of CS sequences corresponded to these taxa and >99% of these were *Streptococcus*.

### The urine Microbiota is Less stable than is the Microbiota of the CS

**Figure 7:**
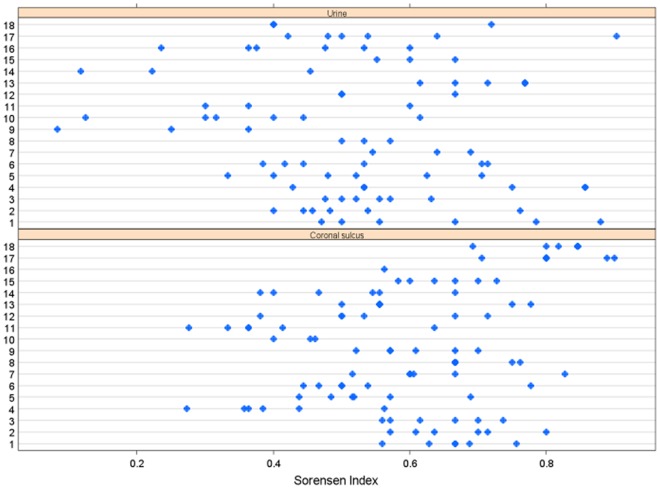
The microbiota of the CS is more stable than that of urine. Sørenson’s similarity indices calculated between pairwise specimens within each participant’s (month = 0, 1, 2, 3) CS and urine samples, separately. A linear mixed-effects model was used to test if the index differed between CS and urine samples, taking into account multiple specimens from the same participants (clustered data). Participants are indicated at left, Sørenson similarity values are on the Y-axis, and blue dots indicate unique specimens.

Similar to CS, independent measures confirmed urine specimens from the same participants were significantly more similar to one another than urine specimens from other participants (Figure 3B). To compare relative stability of the CS and urine microbiotas, Sørensen’s similarity coefficients were computed for pairwise specimens from different sampling points for each subject swab and urine specimen. The analysis incorporated a linear mixed-effect model to account for clustering due to multiple specimens from each subject (Figure 7). Sørensen’s similarity coefficient of CS (0.60) was significantly higher than urine (0.52) (p-value = 0.0001) indicating that the CS microbiota was more stable.

Some urine taxa were stable during the study period (Figures 4B and 5B). Average lengths of continuous colonization with *Propionibacterium* and *Lactobacillus* were 2.33 and 2.75 months respectively, and Lin’s concordance correlation coefficent for these taxa were >0.5. Outside these “core” taxa, the urine microbiota was less stable than CS. *Corynebacteria*, *Anaerococcus, Staphylococcus* and *Prevotella* accounted for only 22.1% of enrollment urine sequences as opposed to 63.5% of CS sequences (Figure 2). Average lengths of continuous colonization with these taxa were all <1.5 months and Lin’s concordance correlation coefficents were all <0.5 (Figure 4B). When present in sequential urine from the same participant the relative abundance of these taxa also varied widely (data not shown). Our interpretation is that some of these taxa may not be true urethral residents but contaminants from the urethral meatus or CS.

### The Microbiota of Urine is Similar to that of other Mucosal Surfaces

Additional results supported the hypothesis that microbial communities of the urethra and CS are distinct. *Lactobacillus*, *Veillonella*, *Aerococcus*, *Ureaplasma*, *Gardnerella* and *Mycoplasma* were detected only, or almost exclusively, in urine. Bacteria in these genera have been identified in mucosal samples of which *Ureaplasma*, *Gardnerella* and *Mycoplasma* are urogenital pathogens [27], [28].

### Partnered sexual exposures impact the penis microbiota

Comparison of the microbiotas of young men in this study with older men from previous studies [18], [19], [20] suggested sexual history could be a determinant of the penis microbiota composition. Known sexually transmitted organisms (*N. gonorrhoeae*, *C. trachomatis*, *U. parvum* and *M. genitalium*) and other poorly characterized taxa associated with the urogenital tract in other studies (*M. hominis* and *Sneathia spp*.) were rare or not detected in this study. Because different types of sexual activities expose the CS and urethra to different microbiotas we reasoned that they might also impact penis microbiotas differently.

Only one participant reported any history of insertive anal sex, whereas 11/18 (61%) reported ever having had vaginal sex and 10/18 (56%) reported fellatio. *Lactobacillus*, *Prevotella*, *Streptococcus, Staphylococcus* and *Anaerococcus* were detected at similar frequencies in urine and CS from participants with and without sexual experience. BV-associated taxa including *Atopobium*, *Megasphaera*, *Mobiluncus*, *Prevotella*, *Gemella*, *Veillonella, Gardnerella* and *Clostridium* [29] were each detected in one or more participants who reported no partnered sexual experiences (Tables S1, S2, S3, S4). These results indicate many putative vaginal taxa also colonize the male urethra and CS, possibly from other body sites or environmental reservoirs. Alternatively, urine from male infants frequently contain *Lactobacillus* [30] so these could be vertically inherited communities. In either scenario, the observation that the male and female genital tracts support similar microbiotas suggests that some of these organisms could survive sexual transfer.

Colonization rates in asymptomatic older men at high risk for STI with various *Ureaplasma*, *Mycoplasma* and *Sneathia* spp. can exceed 30% [18], [19]. In this study, *Ureaplasma*, *Mycoplasma*, and *Sneathia* were each detected in two participants (considering all sets of sequence data and excluding singlets in 454 data-sets that could result from rare miscalls of primer bar codes [31]) and one participant was co-colonized with all three organisms. All of these participants reported sexual exposures. Interestingly, *Sneathia* was detected in one participant co-incident with his first anal sexual exposure. These observations support the hypothesis that sexual exposures could alter the composition of the male urethral flora.

### Similarities of urine and vaginal microbiota

Only 2 enrollment urines lacked *Lactobacillus* and/or *Streptococcus* entirely. One contained a high proportion of unclassified (20.0%) and *Escherichia coli* (36.9%) sequences, indicating possible urinary tract infection. The other contained a mixture of *Prevotella* and *Staphylococcus*. All other enrollment urine contained high proportions of *Streptococcus* or *Lactobacillus,* but rarely both. Spearman’s rank correlation coefficient was used to assess the co-occurrence of taxa in urine specimens. The occurrences of *Lactobacillus* and *Streptococcus* in urine specimens were negatively correlated (ρ = −0.40, p-value = 0.0012). Interestingly, similar *Lactobacillus*- and *Streptococcus*-dominated communities can maintain the low pH of the vagina [32].

Our results show that the penis does not support a single characteristic microbiota and that it is important to distinguish between the urethra and CS in understanding the compositions and possible interactions of these microbiotas with STI. Sexually transmitted pathogens that colonize the penis can exclusively colonize the urethra (*C. trachomatis*, *N. gonorhoeae*, *M. genitalium and U. urealyticum*) or both the urethra and CS (human papilloma virus, herpes simplex virus (HSV-2), human immunodeficiency virus (HIV), *H. ducreyi, T. pallidum*). Whether the CS and urethral microbiota contribute to STI colonization resistance is unclear but reports that circumcision differentially impacts risk for HIV [33], HSV-2, *T. pallidum* and *H. ducreyi* [34] and urethral pathogens (*C. trachomatis* and *N. gonorhoeae*) [35] is in concordance with our findings. A future direction would be to test if the urethral microbiota is relevant to risk for urethral STI.

It has long been appreciated that taxa that resemble those in the female genital tract, colonize the male urethra, at least episodically [21]. Our results are the first to show that some of these taxa persist and colonize the penis prior to the onset of partnered sexual activity. Colonization of the female and male genital tracts with similar bacteria may reflect shared embryonic origins of these tissues. It also suggests that these microbiotas might have common ecological functions, such as lactic acid production and pathogen resistance. A caveat is that 16 S rRNA sequencing does not yield sufficient resolution to differentiate if the taxa in male and female genital tract specimens are indeed the same organisms or tell us if they have similar metabolic capacities.

Finally, our results suggest that partnered sexual behavior can alter the composition of the urogenital microbiota. Although small sample size limits the confidence of some conclusions, it is still noteworthy that BV associated taxa including *Mycoplasma*, *Ureaplasma*, and *Sneathia* were detected only in sexually experienced participants [29]. Technologies that differentiate strain-level polymorphisms, such as metagenomic sequencing, could be used to test if these organisms are sexually transmitted and could yield insights into the nature of idiopathic urogenital tract syndromes that impact both men and women.

## Methods

### Study Design

Young men (ages 14–17 at enrollment; N = 18) were enrolled as a single cohort during the week of January 2, 2010. The participants were a non-clinical sample, recruited from diverse community contacts, although not all are resident in a single community. Additional enrollment criteria included no antibiotic use in the past 60 days, absence of existing urinary tract infection (including sexually transmitted infections), absence of immune suppressing conditions, and overall good general health status. Prior sexual activity was not considered. Self-reported race/ethnicity was White (7/18), Black (7/18), and Latino (4/18).

Self-reported behavioral data was collected at enrollment using a web-based, computer-assisted self-interview. Participants reported circumcision status, lifetime and recent sexual activity (oral, anal, vaginal), and presence or absence of genitourinary symptoms. All participants were asymptomatic at enrollment. All participants and parents provided written, informed consent.

Ethics approval for this study was obtained from the Institutional Review Board of Indiana University and all subjects were recruited through the Indiana University Medical School. Written informed consent to participate in the study was provided by next of kin, caregivers or guardians on the behalf of the minors/children participants in this study and this procedure was approved by the Institutional Review Board of Indiana University.

Specimens were collected at enrollment, and at three subsequent one-month intervals. Specimens were collected in the participant’s home. Voided urine sample and a CS swab sample were collected at each time point. Urine was obtained by first-catch void into a sterile collection cup. CS samples were obtained following training on a flaccid penis model. Participants were instructed to retract the foreskin (if present), and firmly trace the groove of the coronal sulcus circumferentially (using a flocked 4 mm flexible handle elution swab). Samples were immediately placed in a cooler, and transported to the laboratory within four hours were they were stored at −80 C until DNA extraction.

### Molecular Methods

CS samples were thawed and then suspended in 1 ml PBS then were vortexed for 1 min. Urine (10 ml) were thawed and pelleted by centrifugation for 15 min at 4,000×*g* at 4°C. In both cases, DNA was harvested from specimens using a Qiagen DNeasy (Qiagen Inc., Valencia CA) blood and tissue extraction kit. Genomic DNA was eluted in nuclease-free water and stored at 4°C until sequencing. Reagent only controls were processed in parallel to monitor for reagent contamination [18]. 16 S rRNA PCR, Sanger sequencing and assembly of sequences were performed as described previously [19]. Pyrosequencing of the V1–V3, V3–V5 and V6–V9 sub-regions was performed according a protocol developed by the Human Microbiome Project Data Generation Working Group available at http://www.hmpdacc.org [22].

### Sequence Analysis and Statistics

16 S sequences were processed as described previously (18, 19). Briefly, raw 16 S sequence reads were compared to human genome sequences at NCBI using BLASTN [36]. Sequence reads below the BLAST E-value cutoff 10^–20^ were considered putative human DNA contaminants and were removed from the subsequent analyses. For 16 S rRNA contigs obtained from Sanger sequence reads, we applied the GreenGenes criteria to define full-length or near-full 16 S gene (i.e., the sequence length > = 1250 bp). Only full length or near-full length contigs were selected for subsequent analysis. These contigs were screened for chimeras using Bellerophon [37] and default parameters at the GreenGenes web server [38]. For sequence reads obtained from 454 sequencing technology, each read was sorted to specimens only if a perfect barcode sequence was detected. Sequences that did not contain perfect matches to primer barcodes, were less than 200 bp in length, and or had average quality scores of less than 25 were discarded. The primer and barcode sequences were then trimmed from the remaining sequences using customized Perl scripts. Both the 16 S contigs from Sanger sequencing and 16 S raw sequences were classified with RDP Classifier v2.2 [39] using a series of confidence cutoffs ranging from 0.6 to 0.9. For selected subset of genera member sequences were BLASTed against the SILVA database [40]. Species-level assignments were decided based on near perfect alignments to top database matches and were manually examined for alignment quality (i.e., both alignment length and percentage similarity). Multiple sequence alignments were produced using ClustalW [41] with default parameters, and neighbor-joining phylogenic trees were constructed using PHYLIP [42]. Principal coordinate analyses of microbial communities were performed using Unifrac [24]. The above Unifrac analyses were also repeated by random sub-sampling from each specimen. Briefly, sequence reads were randomly extracted without replacement from each specimen (100 reads for Sanger sequences and 200 reads for 454 sequences) and this procedure was repeated 100 times. The averaged sequence counts were used for Unifrac analyses. Microsoft EXCEL and customized scripts developed in the R statistical package (http://www.r-project.org/) were used for statistical analyses. To correct for multiple tests, false discovery rates were computed with the R package function qvalue. Wilcoxon's rank sum test was used to assess comparisons of continuous variables between groups. The Wilcoxon's signed rank test for paired data was used for comparisons between swab and urine samples from the same subject. Fisher’s exact test was used for comparisons of categorical variables between groups. Sørensen’s similarity index was calculated between either swab or urine specimens from different time points within each subject to assess intra-subject variability of microbiotas over time. Differences in the index between swab and urine samples were assessed using linear mixed-effects models. Lin’s concordance correlation coefficent was used to assess the agreement of measurements of abundance of different taxa over time. A 95% confidence interval was also calculated for Lin’s concordance correlation coefficient. Spearman’s correlation coefficent was employed to assess whether the abundances of two taxa were independent or associated. P values less than 0.05 were considered significant. All sequences from this study are available at www.microbiota.org/mum.htm.

## Supporting Information

Figure S1 **Heirarchical clustering of enrollment urine and CS swab microbiomes.** Sanger sequences from enrollment urine and swab specimens were heirarchically clustered using A) Bray-Curtis and B) Spearman’s correlation coefficients as a measure of distance. Urine and swab specimens are labeled U and S, respectively.
(TIF)

Table S1 **RDP classifier summary of 16 S rRNA sequences and select meta-data.** Tables list 90% confidence RDPII results for quality-checked sequences. Sanger data set.
(XLS)

Table S2 **RDP classifier summary of 16 S rRNA sequences and select meta-data.** Tables list 90% confidence RDPII results for quality-checked sequences. V1–V3 data-set.
(XLS)

Table S3 **RDP classifier summary of 16 S rRNA sequences and select meta-data.** Tables list 90% confidence RDPII results for quality-checked sequences. V3–V5 data-set.
(XLS)

Table S4 **RDP classifier summary of 16 S rRNA sequences and select meta-data.** Tables list 90% confidence RDPII results for quality-checked sequences. V6–V9 data-set.
(XLS)

Table S5 **Numbers and sizes of 16 S rRNA amplicons by sample type and method.**
(DOC)
