# Supplementary figures and images for: Bacterial Communities of the Coronal Sulcus and Distal Urethra of Adolescent Males
Source: PLoS One. 2012 May 11;7(5):e36298. doi: 10.1371/journal.pone.0036298 (PMC3350528; doi:10.1371/journal.pone.0036298)

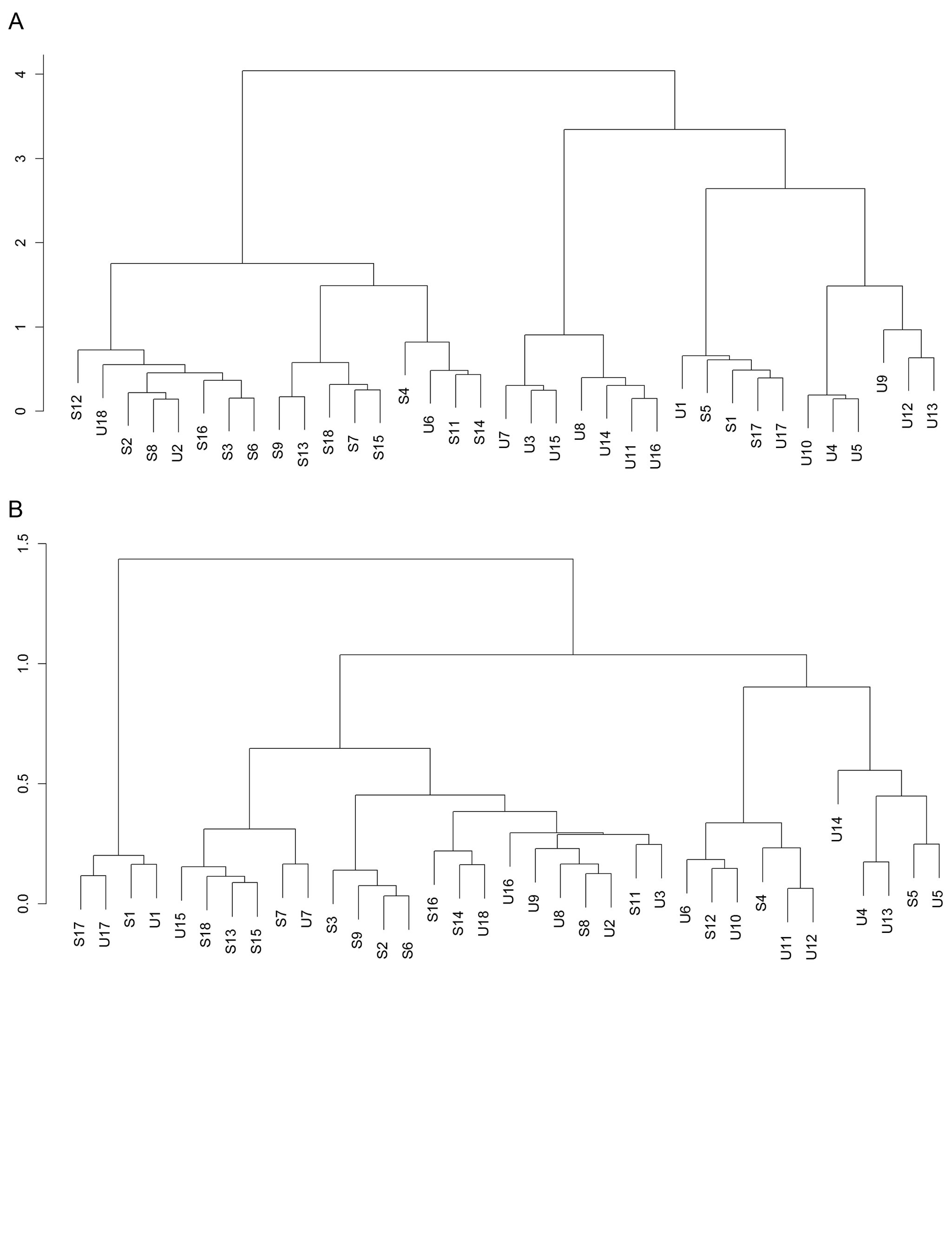

Supplement: Figure S1 — Heirarchical clustering of enrollment urine and CS swab microbiomes. Sanger sequences from enrollment urine and swab specimens were heirarchically clustered using A) Bray-Curtis and B) Spearman’s correlation coefficients as a measure of distance. Urine and swab specimens are labeled U and S, respectively. (TIF) [file pone.0036298.s001.tif]
