# Supplementary material for: Bacterial Communities of the Coronal Sulcus and Distal Urethra of Adolescent Males
Source: PLoS One. 2012 May 11;7(5):e36298. doi: 10.1371/journal.pone.0036298 (PMC3350528; doi:10.1371/journal.pone.0036298)
Supplement: Table S5 — Numbers and sizes of 16 S rRNA amplicons by sample type and method. (DOC) [file pone.0036298.s006.doc]

| Sequence type | Average bp | Average # Reads |
| --- | --- | --- |
| Urine | | |
| Sanger | 1505 +/- 18 | 435 +/- 145 |
| V1-V3 | 459 +/- 65 | 4777 +/- 3818 |
| V3-V5 | 471 +/- 88 | 4503 +/- 4002 |
| V6-V9 | 457 +/- 80 | 8790 +/- 4743 |
| CS | | |
| Sanger | 1493 +/- 14 | 413 +/- 142 |
| V1-V3 | 455 +/- 64 | 7269 +/- 6017 |
| V3-V5 | 474 +/- 84 | 6541 +/- 4581 |
